# Length of hospital stay and associated treatment costs for patients with susceptible and antibiotic-resistant Salmonella infections: a systematic review and meta-analysis

**DOI:** 10.1136/bmjopen-2024-092494

**Published:** 2025-06-23

**Authors:** Chaelin Kim, Isabel Frost, Nichola R Naylor, Heidi Au, Yeonhee Kim, Yubin Lee, Anna Bzymek, Kamila Majgier, Ana Laura Moldoveanu, Omar Mukhtar Salman, Shillah Simiyu, Dina Mohamed Youssef, Mateusz Hasso-Agopsowicz, Kaja Abbas

**Affiliations:** 1 Department of Infectious Disease Epidemiology and Dynamics, London School of Hygiene & Tropical Medicine, London, UK; 2 Department of Immunization, Vaccines and Biologicals, World Health Organization, Geneva, Switzerland; 3 HCAI, Fungal, AMR, AMU & Sepsis Division, UK Health Security Agency, London, UK; 4 Department of Health Services Research and Policy, London School of Hygiene & Tropical Medicine, London, UK; 5 Independent Scholar, London, UK; 6 College of Medicine, Dankook University, Cheonan, Korea (the Republic of); 7 Faculty of Pharmacy, Wroclaw Medical University, Wroclaw, Poland; 8 Consultant, World Health Organization, Geneva, Switzerland; 9 Imperial College London, London, UK; 10 School of Tropical Medicine and Global Health, Nagasaki University, Nagasaki, Japan; 11 Institute of Tropical Medicine, Nagasaki University, Nagasaki, Japan; 12 Public Health Foundation of India, New Delhi, India

**Keywords:** Public health, Economics, INFECTIOUS DISEASES

## Abstract

**Abstract:**

**Objectives:**

The global disease burden of *Salmonella* infections in 2017 included 135 900 deaths caused by *Salmonella* Typhi and Paratyphi and 77 500 deaths caused by invasive non-typhoidal *Salmonella*, with increasing antimicrobial resistance (AMR) exacerbating morbidity, mortality and costs. The aim of our systematic review and meta-analysis is to estimate the length of hospital stay and associated treatment costs for patients with susceptible and antibiotic-resistant *Salmonella* Typhi, Paratyphi and non-typhoidal *Salmonella* infections.

**Design:**

Systematic review and meta-analysis.

**Data sources:**

We searched EMBASE, Medline/PubMed, Scopus, Hinari and LILACS databases for studies published between 1 January 2005 and 15 May 2024, with no language restrictions.

**Eligibility criteria:**

We included 30 studies that reported the length of hospital stay or treatment costs for patients with susceptible or antibiotic-resistant *Salmonella* Typhi, Paratyphi and non-typhoidal *Salmonella* infections. We excluded studies with sample sizes of less than 30 patients, those focused on non-human subjects and those not reporting our outcomes of interest.

**Data extraction and synthesis:**

Two reviewers independently screened studies and extracted data on the length of hospital stay and associated costs, with monetary values converted to 2019 USD. We aggregated data according to GDP per capita quantiles using a random-effects meta-analysis. We conducted a quality assessment using an adapted Joanna Briggs Institute tool.

**Results:**

Patients with drug-resistant *Salmonella* infections had longer hospital stays, with an additional 0.5–2.2 days compared with drug-susceptible *Salmonella* infections. Based on our meta-analysis, the mean hospital stay for typhoidal *Salmonella* infections was 6.4 days (95% CI 4.9 to 7.8) for drug-susceptible cases and 8.4 days (95% CI 5.1 to 11.7) for resistant cases in the lowest income quartiles. While there were insufficient data to perform a pooled analysis, individual studies inferred that treatment costs for resistant typhoidal *Salmonella* infections were higher than for susceptible infections, and resistant non-typhoidal *Salmonella* infections had longer hospital stays and higher costs compared with susceptible infections. Data were scarce from high-*Salmonella*-burden countries, particularly in sub-Saharan Africa and parts of Asia.

**Conclusions:**

Patients with antibiotic-resistant *Salmonella* infections experience a greater healthcare burden in terms of hospitalisation length and direct costs compared with those with susceptible infections. We highlight the economic burden of AMR in *Salmonella* infections and emphasise the need for preventive measures.

**STRENGTHS AND LIMITATIONS OF THIS STUDY**

- This study used a systematic review and meta-analysis approach, adhering to Preferred Reporting Items for Systematic Reviews and Meta-Analyses guidelines, to synthesise data on hospitalisation length and treatment costs associated with *Salmonella* infections.
- Inclusion of multiple databases with no language restrictions reduced selection bias and improved the comprehensiveness of the search strategy.
- The use of GDP per capita quantiles to stratify cost data provides a contextual understanding of economic burden across different income settings.
- A key limitation is the scarcity of data from regions with high *Salmonella* burden, particularly in sub-Saharan Africa and parts of Asia.
- Variability in study designs, definitions of drug resistance and incomplete reporting of cost data across studies may have introduced heterogeneity into the meta-analysis.

## Introduction

*Salmonella* spp are gram-negative bacteria that cause foodborne illnesses and are classified into two species: *Salmonella enterica* and *Salmonella bongori. Salmonella enterica* subspecies *enterica* includes both typhoidal and non-typhoidal *Salmonella* (NTS). Typhoidal *Salmonella* refers to serovars Typhi and Paratyphi (A, B and C) that cause enteric fever. NTS refers to the other serovars (e.g., Typhimurium and Enteritidis,) that primarily cause gastroenteritis and invasive diseases.^1 2^

The primary mode of *Salmonella* transmission is through the faecal-oral route via contaminated food and water. While infections caused by various *Salmonella* serovars can manifest as mild and self-limiting conditions, they can progress to severe symptoms that may require hospitalisation.^3^ NTS, in particular, can cause febrile invasive disease (iNTS), which has a high case fatality rate of 20%–28% among children in Africa.^4^ Enteric fever caused by *Salmonella* Typhi and *Salmonella* Paratyphi accounted for 135 900 deaths globally in 2017.^5^ There were also an estimated 5 35 000 cases of iNTS worldwide in the same year, leading to around 77 500 deaths.^6^

The increasing antibiotic resistance in *Salmonella enterica* serovars threatens the effective treatment of *Salmonella* infections, with high resistance rates reported in various regions.^7^ In South Asia, overall antimicrobial resistance (AMR) in *Salmonella* increased from 53% to 77% over 10 years.^8^ In South America, AMR in *Salmonella* increased significantly between 2012 and 2021, with resistance rates to key antibiotics such as ciprofloxacin, ampicillin, ampicillin/sulbactam and ceftriaxone. In particular, ciprofloxacin resistance in *Salmonella* isolates increased from negligible levels in 2012–2013 to up to 60% in 2020–2021.^9^

*Salmonella* Typhi isolates are classified as multidrug-resistant (MDR) when they are resistant to chloramphenicol, ampicillin and trimethoprim-sulfamethoxazole and as extensively drug-resistant (XDR) when they meet the MDR criteria and are also resistant to fluoroquinolones and third-generation cephalosporins.^10^,^12^ The emerging resistance has rendered first-line antibiotics ineffective for treating *Salmonella* infections, while resistance to these critical second-line treatments, such as fluoroquinolones and cephalosporins, is also on the rise. In *Salmonella* Typhi, resistance markers were found in 100% of sequenced isolates from Pakistan and 75%–97% from Nepal, Cambodia and India. For NTS, fluoroquinolone resistance rates varied by serovar, country and source, with up to 13% of Typhimurium isolates from Scotland and 7% from the USA showing resistance.^13^

With rising AMR in *Salmonella*, assessing the health and economic burden and planning strategic responses are essential. Patients with drug-resistant *Salmonella* infections have excess mortality compared with those with susceptible infections.^14 15^ Additionally, they are associated with severe disease that results in an excess risk of hospitalisation, longer length of hospital stays and increased hospital costs compared with patients with susceptible *Salmonella* infections.^16 17^ Although individual studies have explored these outcomes in particular contexts, there has yet to be a comprehensive systematic review that synthesises the global economic burden of drug-resistant *Salmonella* infections.

Therefore, we conducted a systematic review and meta-analysis to estimate the length of hospital stay and associated treatment costs for patients with susceptible or antibiotic-resistant *Salmonella* Typhi, Paratyphi and NTS infections. Our specific study questions examine two key areas: (1) morbidity, utility and productivity impacts (Q1–Q2) and (2) hospital and healthcare system costs (Q3–Q6). Specifically, we sought to answer the following questions: (Q1) What is the length of symptomatic infection with a drug-susceptible, mixed or unknown resistant *Salmonella* infection? (Q2) What is the length of symptomatic infection with a drug-resistant *Salmonella* infection? (Q3) What is the length of hospitalisation with a drug-susceptible, mixed or unknown resistant *Salmonella* infection? (Q4) What is the length of hospitalisation with a drug-resistant *Salmonella* infection? (Q5) What is the per-patient cost of infection with a drug-susceptible, mixed or unknown resistant *Salmonella* infection? (Q6) What is the per-patient cost of an infection with a drug-resistant *Salmonella* infection?

## Methos

### Search strategy

We adhered to the PRISMA (Preferred Reporting Items for Systematic Reviews and Meta-Analyses) guidelines for reporting our systematic review and meta-analysis (see online supplemental material 1).^18^ We searched the EMBASE, Medline/PubMed, Scopus, Hinari and LILACS databases for articles published between 1 January 2005 and 15 May 2024, with no language restrictions (see online supplemental material 2A for the search strategy). For relevant publications in languages not known to the authors, we used Google Translate tools to translate abstracts and full-text papers to assess their eligibility and extract data. We selected 2005 as the start date to ensure that diagnoses and treatments for *Salmonella* infections reflect current clinical standards and that reported costs can meaningfully inform our analyses. We conducted our search in two stages. First, we searched for articles published between 1 January 2005 and 17 December 2021, and then we updated our search to cover the period between 15 December 2021 and 15 May 2024. The same search strategy was consistently applied throughout both stages of the search to ensure continuity.

Eligible study populations include those infected with antimicrobial-resistant and/or susceptible bacteria: typhoidal *Salmonella* (*Salmonella enterica* Typhi and Paratyphi) and NTS. Our inclusion and exclusion criteria were based on the population, intervention, comparison, outcome framework (see online supplemental material 2B).

### Study selection

Eight reviewers (CK, YK, AB, KM, ALM, OMS, SS and DMY) screened the articles. Two reviewers independently screened each study’s title and abstract to determine eligibility, and a third reviewer (IF or MH-A) resolved any discrepancies. After title/abstract screening, we reviewed the full text of eligible articles for study screening and data extraction.

### Data extraction

The eight reviewers manually extracted data using standardised forms within the DistillerSR platform and used Google Sheets for subsequent data management. We extracted the following information from the included studies: author, year of publication, title, country/region, study dates and duration, study design, pathogens analysed, cohort details, detection method, length of hospital stay, and cost of hospitalised treatment and hospital stay. Where available, we extracted data on community cost indicators (e.g., length of illness) and comparisons of outcomes between drug-sensitive and resistant pathogens.

### Quality assessment

Two of three reviewers (CK, YL and HA) independently performed the quality assessments for each study, and discrepancies were resolved through discussion. We assessed the quality of the included studies using an adapted version of the Joanna Briggs Institute critical appraisal tool, using the quality assessment checklist specific to each study design.^19^

Since there is no checklist for cost-of-illness studies, which focus on the economic burden of diseases rather than comparing interventions, we adapted the economic evaluation checklist to assess the cost-of-illness study design (online supplemental material 2C). We estimated the risk of bias assessment outcome for each included study and calculated average quality scores for the studies grouped by their respective study design.

### Statistical analysis

We categorised the extracted data on length of symptomatic infection, length of hospitalisation and per-patient costs according to different types of *Salmonella* enterica: *Salmonella* Typhi, *Salmonella* Paratyphi and NTS and their resistance status. For all extracted data, including the number of days and costs, we converted any estimates in other measures (ie, median) and uncertainty/range values (ie, SD, range, IQR) into means and 95% CIs to make results comparable across studies.^20^ To account for economic differences between countries, we categorised them into quartiles based on their gross domestic product (GDP) per capita using 2019 World Bank data.^21^ We calculated quantile cut-offs by dividing the GDP per capita values into four equal groups (quartiles), where quantile-1 represented the lowest quartile and quantile-4 the highest. We performed meta-analyses using a random effects model stratified by these GDP per capita quartiles, including only subcategories with more than three estimates from different study points. This quartile-based approach was chosen to provide more contextually relevant estimates across different economic settings rather than pooling all estimates globally. A random-effects model was chosen to account for anticipated heterogeneity between studies due to differences in study designs, population characteristics and geographical settings. Online supplemental material 2D details how we converted costs into 2019 US dollars and performed the meta-analysis. We conducted our analysis using R software (V.4.2.3), and the code is publicly accessible for reproducible analysis on https://github.com/ckim0509/amr_cost.

### Patient and public involvement

None.

## Results

**Figure 1:**
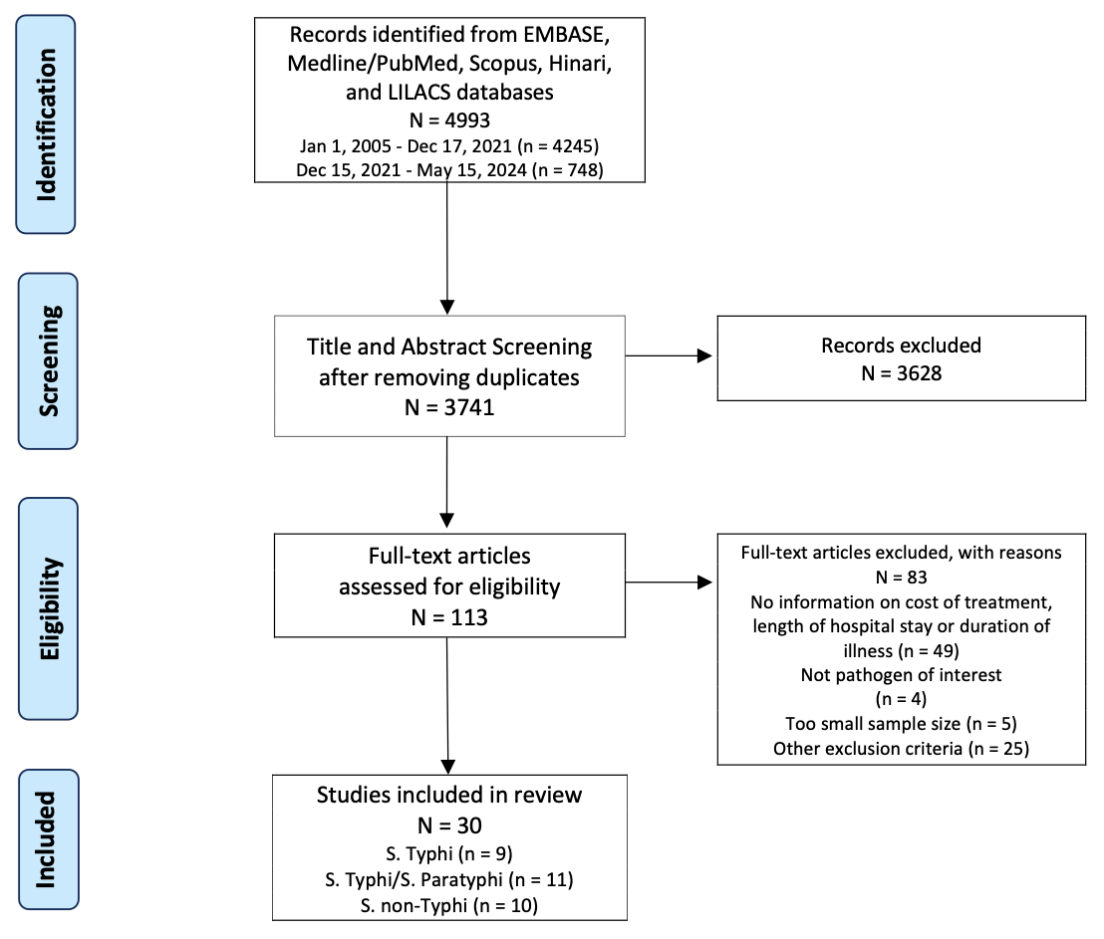
Preferred Reporting Items for Systematic Reviews and Meta-Analyses flow diagram. After removing duplicates from the 4993 studies identified, 3741 studies published between 1 January 2005 and 15 May 2024 were identified. After screening titles and abstracts, 113 studies proceeded to full-text review. After full-text screening, 83 studies were excluded due to different outcomes, different pathogens, small sample sizes and other reasons. 30 publications were ultimately included in the review. *S*. non-Typhi, non-typhoidal *Salmonella*; *S.* Paratyphi, *Salmonella* Paratyphi; *S*. Typhi, *Salmonella* Typhi.

The PRISMA flow diagram illustrates the identification, screening, eligibility and inclusion stages of our systematic review (see figure 1). We streamlined to 30 studies in our systematic review and included studies with populations affected by *Salmonella* Typhi (n=9), *Salmonella* Typhi and *Salmonella* Paratyphi (n=11) and NTS (n=10). The studies were conducted across diverse geographical locations, including Australia (n=1), Bangladesh (n=3), China (n=3), India (n=6), Indonesia (n=1), Italy (n=1), Nepal (n=1), New Zealand (n=1), Pakistan (n=8), Spain (n=1), Sweden (n=1), Taiwan (n=2), Turkey (n=2), the UK (n=1), the USA (n=2) and Vietnam (n=2). In some instances, multiple countries were involved in the same study.^22 23^

The 30 studies included 15 case series studies, 4 cohort studies, 4 cost of illness studies, 2 cross-sectional studies, 4 prevalence studies and 1 randomised controlled trial. The average quality scores were 0.93 for case series studies, 0.83 for cohort studies, 0.91 for cost of illness studies, 0.83 for cross-sectional studies, 1 for prevalence studies and 0.55 for the randomised controlled trial (see table 1).

**Table 1:** Characteristics of studies in the systematic review

Study	Country	Study design	Pathogen	Resistant profile	Study period	Quality score
Aypak *et al*^24^	Turkey	Case series study	*S*. Typhi	MDR cases (resistant to all three first-line antibiotics: ampicillin, cotrimoxazole (TMP-SMX) and chloramphenicol)	2008–2008	0.80
Bandyopadhyay *et al*^38^	India	Cohort study	*S*. Typhi and *S*. Paratyphi	Not reported cases; Nalidixic acid sus.; MDR cases (resistant to nalidixic acid, ampicillin, chloramphenicol, ciprofloxacin and trimethoprim-sulfamethoxazole (TMP-SMZ))	2008–2012	0.60
Björklund *et al*^57^	Sweden	Cohort study	*S*. non-Typhi	Mixed cases	2012–2022	1.00
Broughton *et al*^25^	China	Case series study	*S*. non-Typhi	Nalidixic acid sus.; Nalidixic acid res.	2003–2008	0.90
Dahiya *et al*^39^	India	Case series study	*S*. Typhi and *S*. Paratyphi	Not reported	2013–2016	0.80
Duong *et al*^35^	Vietnam	Prevalence study	*S*. non-Typhi	Mixed cases	2014–2016	1.00
Fatima *et al*^26^	Pakistan	Case series study	*S*. Typhi	Non-XDR; XDR (resistant to ampicillin, chloramphenicol, TMP-SMZ, ciprofloxacin and ceftriaxone)	2016–2018	1.00
Ganesh *et al*^58^	India	Case series study	*S*. Typhi	Mixed cases	2005–2008	1.00
Garrido-Estepa *et al*^43^	Spain	Prevalence study	*S*. non-Typhi	Not reported	2010–2015	1.00
Herekar *et al*^27^	Pakistan	Case series study	*S*. Typhi	Drug-sensitive; MDR cases (resistant to ampicillin, TMP-SMZ, and chloramphenicol); XDR cases (MDR+resistant to fluoroquinolones)	2017–2018	0.89
Huang *et al*^59^	Taiwan	Case series study	*S*. non-Typhi	Mixed cases	2005–2009	1.00
Hume *et al*^28^	Australia	Case series study	*S*. Typhi and *S*. Paratyphi	Mixed cases; Nalidixic acid susceptible cases; Nalidixic acid resistant cases	1990–2007	1.00
Karakecili *et al*^29^	Turkey	Case series study	*S*. Typhi	Amikacin-resistant (still susceptible to ampicillin, ceftriaxone, and ciprofloxacin).	–	1.00
Khan *et al*^36^	Pakistan	Cross-sectional study	*S*. Typhi	MDR cases; XDR cases	2021–2021	0.83
Khatun *et al*^60^	Bangladesh	Cohort study	*S*. Typhi and *S*. Paratyphi	Mixed cases	2010–2014	0.86
Lane *et al*^61^	New Zealand	Case series study	*S*. Typhi and *S*. Paratyphi	Mixed cases	2005–2010	1.00
Lee *et al*^40^	Taiwan	Cohort study	*S*. non-Typhi	Mixed cases	2010–2018	0.86
Liang *et al*^62^	China	Prevalence study	*S*. non-Typhi	Mixed cases	2014–2016	1.00
Longley *et al*^22^	Nepal, Bangladesh, Pakistan	Case series study	*S*. Typhi and *S*. Paratyphi	Mixed cases	2016–2019	1.00
Mahmood *et al*^63^	Pakistan	Cross- setional study	*S*. Typhi	XDR cases	2022–2022	0.83
Mejia *et al*^41^ (1)	Pakistan	Cost of illness study	*S*. Typhi and *S*. Paratyphi	Mixed cases; MDR cases; XDR cases	2016–2018	1.00
Mejia *et al*^42^ (2)	Bangladesh	Cost of illness study	*S*. Typhi and *S*. Paratyphi	Not reported	2016–2018	1.00
Mukherjee *et al*^31^	USA	Prevalence study	*S*. non-Typhi	Pansusceptible; Resistant cases; Tetracycline susceptible; Tetracycline resistant cases; Ampicillin susceptible; Ampicillin resistant cases	2011–2014	1.00
Nagaraj *et al*^64^	India	Randomised controlled trial	*S*. Typhi and *S*. Paratyphi	Not reported	2013–2014	0.55
Pagani *et al*^37^	Italy	Case series study	*S*. non-Typhi	Mixed cases	2015–2021	1.00
Poulos *et al*^23^	Vietnam, China, Indonesia, Pakistan, India	Cost of illness study	*S*. Typhi	Not reported	2002–2005	0.90
Reddy *et al*^32^	UK	Cost of illness study	*S*. Typhi and *S*. Paratyphi	Ciprofloxacin susceptible; Ciprofloxacin resistant cases	2005–2010	0.75
Shahid *et al*^33^	Pakistan	Case series study	*S*. Typhi	XDR cases (resistance to the five classes of antibiotics (ampicillin, chloramphenicol, TMP-SMZ, fluoroquinolones and third generation cephalosporin (ceftriaxone or cefixime))	2017–2018	1.00
Sharma *et al*^65^	India	Case series study	*S*. Typhi and *S*. Paratyphi	Mixed cases	2014–2019	0.75
Solghan *et al*^34^	USA	Case series study	*S*. non-Typhi	Pansusceptible, MDR (resistance to at least ampicillin, chloramphenicol, streptomycin, sulfisoxazole and tetracycline).	2003–2007	0.78

Characteristics of included studies in the systematic review and meta-analysis to estimate the length of hospital stay and associated treatment costs for patients with susceptible or antibiotic-resistant *Salmonella* Typhi, Paratyphi and non-typhoidal *Salmonella* infections.

MDR, multidrug-resistant; S. non-Typhi, non-typhoidal *Salmonella*; S. Paratyphi, *Salmonella* Paratyphi; S. Typhi, *Salmonella* Typhi; XDR, extensively drug-resistant.

### Length of hospital stays

**Figure 2:**
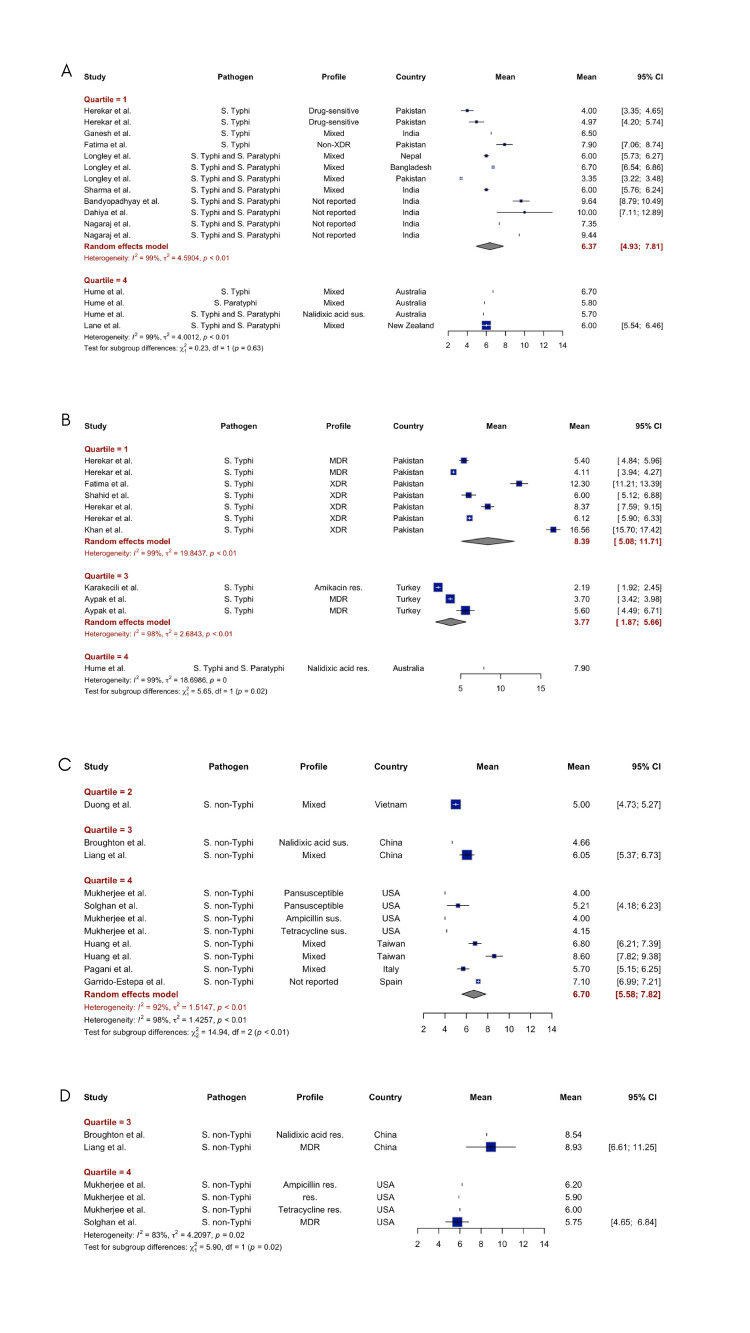
Length of hospital stay for drug-susceptible and drug-resistant *Salmonella* Typhi, Paratyphi and non-typhoidal *Salmonella* infections. The length of hospital stays for drug-susceptible and drug-resistant *Salmonella* Typhi, Paratyphi and nontyphoidal *Salmonella* infections was classified by GDP per capita quantile level and estimated from the meta-analysis. (**A**) Length of hospital stays of susceptible *Salmonella* Typhi and/or Paratyphi infection. (**B**) Length of hospital stays of resistant *Salmonella* Typhi and/or Paratyphi infection. (**C**) Length of hospital stays of susceptible *Salmonella* non-Typhi infection (**D**) Length of hospital stays of resistant *Salmonella* non-typhi infection. GDP, gross domestic product; MDR, multidrug-resistant; *S*. non-Typhi, non-typhoidal *Salmonella*; *S.* Paratyphi, *Salmonella* Paratyphi; *S*. Typhi, *Salmonella* Typhi; XDR, extensively drug-resistant.

There were 13 studies^24^,^36^ that investigated the length of hospital stay for patients infected with resistant *Salmonella enterica* strains, while 7 of them^25 27 28 31 32 34 37^ also reported the length of stay for patients with drug-susceptible *Salmonella enterica* strains. The included studies showed patterns of longer hospital stays for patients with resistant *Salmonella* infections compared with those with susceptible infections across different types of *Salmonella enterica* and economic settings. For typhoidal *Salmonella* (*Salmonella* Typhi and *Salmonella* Paratyphi) in the lowest income quantile (Q1), our pooled estimate showed a mean hospital stay of 6.4 days (95% CI 4.9 to 7.8 days) for drug-susceptible, mixed or unknown resistant infections. In contrast, resistant typhoidal *Salmonella* infections in the same economic setting had a significantly longer mean stay of 8.4 days (95% CI 5.1 to 11.7 days), representing a 31% increase in hospitalisation duration. For NTS in high-income countries (Q4), our meta-analysis showed a mean hospital stay of 6.7 days (95% CI 5.6 to 7.8 days) for susceptible infections. We had insufficient data to generate pooled estimates for resistant infections (see figure 2 and online supplemental material 2E).

Related studies have also supported pooled findings. In the study by Hume *et al*,^28^ patients with enteric fever caused by nalidixic acid resistant *Salmonella* had a significantly longer mean hospital stay of 7.9 days compared with 5.7 days for patients infected with non-nalidixic acid resistant isolates, representing a 2.2 day longer hospitalisation on average associated with nalidixic acid resistant strains. In a study by Herekar *et al*,^27^ patients with XDR *Salmonella* Typhi infections had the most prolonged median hospital stay of 8 days for adults and 6 days for children, compared with patients with MDR infections (median 5 days for adults and 4 days for children) and drug-sensitive infections (median 4 days for adults and 4.5 days for children).

Among patients with NTS, the mean hospital stay was significantly longer for those with antibiotic-resistant isolates compared with fully susceptible isolates (5.9 vs 4 days, p<0.05). Specifically, tetracycline resistance was associated with a 6 vs 4.2 day stay (p=0.068), while ampicillin resistance was associated with a 6.2 vs 4 day stay (p<0.05).^31^ Furthermore, a study by Duong *et al*^35^ in Vietnam reaffirmed that NTS patients resistant to more than one antibiotic had a pairwise mean difference of 0.91 extra hospital days (p=0.04) compared with fully susceptible isolates. In this study, ceftriaxone resistance significantly prolonged hospitalisation in children initially treated with third-generation cephalosporins (Wilcoxon signed-rank test; p ≤0.02), while ciprofloxacin resistance was not associated with longer length of stay for patients initially receiving fluoroquinolones in comparison to drug-susceptible patients. Patients admitted to a Hong Kong hospital with NTS infections resistant to nalidixic acid (indicating quinolone resistance) had 33% longer hospital stays compared with patients infected with NTS fully susceptible to quinolones (median of 4 days vs 3 days, p<0.05).^25^ Patients with NTS resistance to ampicillin, chloramphenicol, streptomycin, sulfisoxazole and tetracycline had a median hospital stay of 4.5 days, which was approximately half a day longer than the median stay of 4 days for patients with pan-susceptible NTS isolates.^34^

### Length of illness

**Figure 3:**
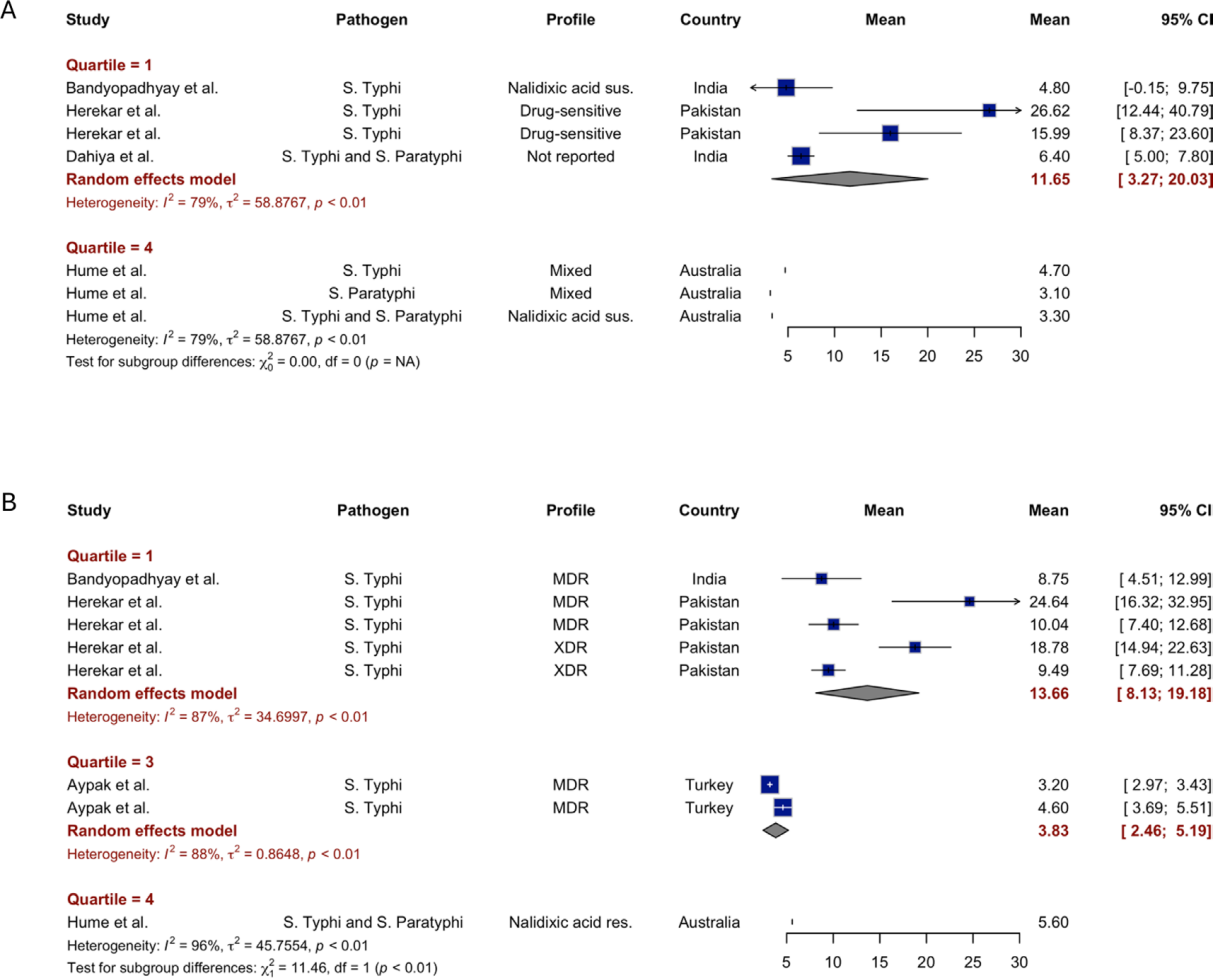
Length of illness for drug-susceptible and drug-resistant *Salmonella* Typhi and Paratyphi. The length of hospital stays for drug-susceptible and drug-resistant *Salmonella* Typhi and Paratyphi infections was classified by GDP per capita quantile level and estimated from the meta-analysis. (**A**) Length of illness (fever) of susceptible *Salmonella* Typhi and/or Paratyphi infection (**B**) Length of illness (fever) of resistant *Salmonella* Typhi and/or Paratyphi infection. GDP, gross domestic product; MDR, multidrug-resistant; *S*. non-Typhi, non-typhoidal *Salmonella*; *S.* Paratyphi, *Salmonella* Paratyphi; *S*. Typhi, *Salmonella* Typhi; XDR, extensively drug-resistant.

Studies have reported the duration of fever based on fever clearance time in patients infected with susceptible^2728 38^,^40^ or resistant^24 27 28 38^*Salmonella enterica* serovars. For fever duration in typhoidal *Salmonella* (*Salmonella* Typhi and *Salmonella* Paratyphi) in the lowest income quantile, our meta-analysis estimated a mean fever clearance time of 11.7 days (95% CI 3.3 to 20 days) for drug-susceptible, mixed or unknown resistant infections. The pooled mean estimate for drug-resistant infections in the same setting was longer at 13.7 days (95% CI 8.1 to 19.2 days) (see figure 3 and online supplemental material 2E).

Related studies showed mixed results regarding fever duration differences between resistant and susceptible strains. Bandyopadhyay *et al*^38^ found that the mean fever clearance times were similar among patients infected with nalidixic acid-susceptible, nalidixic acid-resistant and MDR *Salmonella* Typhi. Similarly, Herekar *et al*^27^ also found no systematic differences in fever duration among patients infected with drug-sensitive, MDR and XDR strains. Khan *et al*^36^ reported that when antibiotics selected on the basis of laboratory-confirmed bacterial susceptibility were administered, the mean time to fever clearance was 4.03 days, significantly shorter than the overall mean of 10.84 days from antibiotic initiation to fever clearance.

### Hospital-related healthcare costs

Our meta-analysis of healthcare costs was constrained by the limited number of studies that reported costs according to resistance status. Nevertheless, the available studies consistently demonstrated higher costs related to resistant infections. Two studies^25 41^ estimated the direct hospital costs of resistant *Salmonella* infections. Mejia *et al*^41^ estimated the costs of overall *Salmonella* Typhi and *Salmonella* Paratyphi for mixed, MDR and XDR cases in Pakistan. The mean direct hospital costs (e.g., registration, clinical examination, inpatient stay, laboratory tests, drugs and medicines) were US$176 for all cases, US$139 for MDR cases and US$281 for XDR cases (all costs in 2019 USD). This represents a 103% increase in costs from MDR to XDR patients. Broughton *et al*^25^ compared medical costs for NTS in Hong Kong between quinolone-susceptible and quinolone-resistant patients. The mean costs were US$3493 and US$5961 for quinolone-susceptible and quinolone-resistant cases respectively, indicating a 71% increase in costs. Additional studies have reported direct hospital costs of *Salmonella* infections without differentiating resistance status.^32 42 43^ Hospitalised patients incurred US$78 in Bangladesh for *Salmonella* Typhi and *Salmonella* Paratyphi,^42^ US$2874 in the UK^32^ and US$5351 in Spain for *Salmonella* non-Typhi.^43^ Table 2 presents hospital costs for patients with susceptible and antibiotic-resistant *Salmonella* infections.

**Table 2:** Hospital costs for patients with susceptible and antibiotic-resistant *Salmonella* infections

Question	Pathogen	Classification	Country	Quantile level of GDP per capita	Sample size	Hospital costs2019 USD (95% CI)*	Reference
What are the per-patient costs of an infection with a drug-susceptible, mixed or unknown-resistant *Salmonella* infection?	*S*. Typhi and *S*. Paratyphi	Mixed	Pakistan	1	980	175.5 (161.8 to 189.3)	Mejia *et al*^41^ (1)
	*S*. Typhi and *S*. Paratyphi	Not reported	Bangladesh	1	1735	78.1 (74.2 to 82)	Mejia *et al*^42^ (2)
	*S*. Typhi and *S*. Paratyphi	Not reported	UK	4	138	2873.6	Reddy *et al*^32^
	*S*. non-Typhi	Nalidixic acid sus.	China	3	163	3492.7	Broughton *et al*^25^
	*S*. non-Typhi	Not reported	Spain	4	21 660	5351.2 (5287.3 to 5415.1)	Garrido-Estepa *et al*^43^
What is the per-patient cost of an infection with a drug-resistant *Salmonella* infection?	*S*. Typhi and *S*. Paratyphi	MDR	Pakistan	1	164	138.8 (110.9 to 166.8)	Mejia *et al*^41^ (1)
	*S*. Typhi and *S*. Paratyphi	XDR	Pakistan	1	387	281.1 (242.5 to 319.6)	Mejia *et al*^41^ (1)
	*S*. non-Typhi	Nalidixic acid res.	China	3	162	5960.9	Broughton *et al*^25^

* Mean values (how other forms of values were transformed to mean and CIs; https://rdrr.io/cran/meta/man/metamean.html.

GDP, Gross domestic product; MDR, multidrug-resistant; S. non-Typhi, Non-typhoidal *Salmonella*; *S*. Paratyphi, *Salmonella* Paratyphi; *S*. Typhi, *Salmonella* Typhi; XDR, extensively drug-resistant.

## Discussion

Our systematic review and meta-analysis of studies estimated the length of hospital stay and associated treatment costs for patients with susceptible or antibiotic-resistant *Salmonella* Typhi, Paratyphi and NTS infections. Our findings show that patients with drug-resistant *Salmonella* infections had longer hospital stays, with an additional 0.5–2.2 days compared with drug-susceptible *Salmonella* infections. Our meta-analysis results show that the mean hospital stay for typhoidal *Salmonella* infections was 6.4 days (95% CI 4.9 to 7.8) for drug-susceptible cases and 8.4 days (95% CI 5.1 to 11.7) for resistant cases in the lowest income quartiles. Individual studies have suggested that treatment costs for resistant typhoidal *Salmonella* infections were higher than for susceptible infections and resistant NTS infections had longer hospital stays and higher costs compared with susceptible infections. Duration of fever varied across studies without clear patterns related to resistance.

Increased costs and hospital stays for resistant *Salmonella* infections are expected, given that resistant infections require more time to clear than susceptible infections, as has been demonstrated for other pathogens.^44^,^46^ This could be explained by increased antibiotic treatment failure for resistant *Salmonella* infections. Duong *et al* showed that changes to secondary or tertiary antimicrobials from the initial primary treatment were significantly correlated with prolonged hospitalisation.^35^

Our systematic review found few primary studies from regions with high typhoid fever burdens, including sub-Saharan Africa, parts of Southeast Asia (e.g., Cambodia, Laos, Myanmar), and the remaining Oceanic countries. Typhoid fever remains highly endemic in these settings. Furthermore, iNTS infections are especially prevalent in sub-Saharan Africa. Despite the high disease burden in these regions, our review found limited research quantifying healthcare costs, hospitalisation duration and economic consequences associated with resistant versus susceptible *Salmonella* infections. Better utilisation of routine healthcare records, including electronic health records where available, will help fill the gaps in data and evidence for *Salmonella*.

Our study has limitations. First, the timing of antibiotic administration, hospital presentation and prehospitalisation antibiotic use have not been controlled in our primary studies and have varied. This would likely not differ between resistant and non-resistant infections, but this might have contributed to the heterogeneity in our meta-analysis outcomes. Similarly, variability in study design and quality might have reduced the strength of the pooled estimates derived from our meta-analysis. The quality of evidence on length of hospital stay and costs varied across the included studies. Many studies were observational without control groups, limiting the ability to attribute outcomes to antibiotic resistance status. In addition, while we have pooled estimates across countries, healthcare costs can vary substantially across different healthcare systems and settings. We combined estimates to gain a broader overview of the economic burden and used random effects models along with gross domestic product (GDP) per capita quantiles to account for between-study heterogeneity and minimise differences in costs across countries. However, there remains uncertainty around applying cost estimates from one context to another.

Quantifying the impact on healthcare utilisation and costs through this systematic approach is critical for informing economic evaluations and policy decisions regarding interventions to curb *Salmonella* infections and AMR. The estimates from our study make a valuable contribution to understanding the overall disease and economic burden of AMR *Salmonella*. Our findings can be incorporated into disease models used for estimating the health and economic impact of interventions (e.g., vaccines, improved diagnostics, and antimicrobial stewardship programmes) on reducing the burden of AMR *Salmonella*.^47 48^

Preventive measures such as improved access to safe water, sanitation and hygiene (WASH) are essential to block transmission pathways.^49^ Further, vaccines mitigate AMR by preventing diseases and reducing antibiotic use associated with infections.^50 51^ WHO recommends introducing and scaling up typhoid conjugate vaccines (TCV) alongside WASH interventions in typhoid-endemic areas.^52^ Pakistan, Liberia and Zimbabwe have included TCV in immunisation programmes and campaigns.^53^ However, TCV introduction to other endemic regions has faced barriers including regulatory processes, development of implementation strategies and competing health priorities.^54^ Furthermore, vaccines are still lacking for *Salmonella* Paratyphi and NTS, although progress is being made to develop new vaccines.^55 56^

Robust data on the health and economic burden of resistant *Salmonella* strains are essential for evaluating the value of these interventions. Our systematic review addresses this critical evidence gap by providing estimates to quantify the healthcare and economic costs of AMR *Salmonella*. Furthermore, our methodological approach establishes a framework that can be applied to other high-priority pathogens where the evidence on the health and economic burden of AMR is limited.

In conclusion, based on our systematic review and meta-analysis, we confirm that resistant strains of *Salmonella* are associated with an increased economic burden in terms of increased hospitalisation costs and length of stay. However, there are remaining gaps in understanding the specific healthcare costs and extended durations of hospitalisation linked to antibiotic resistance in *Salmonella* infections, especially in high-*Salmonella*-burden countries, particularly in sub-Saharan Africa and parts of Asia, and this warrants future studies to address these evidence gaps.

## Supplementary material

10.1136/bmjopen-2024-092494 online supplemental material 1

10.1136/bmjopen-2024-092494 online supplemental material 2

## Data Availability

Data are available in a public, open access repository.
