# Supplementary material for: Length of hospital stay and associated treatment costs for patients with susceptible and antibiotic-resistant Salmonella infections: a systematic review and meta-analysis
Source: BMJ Open. 2025 Jun 23;15(6):e092494. doi: 10.1136/bmjopen-2024-092494 (PMC12186044; doi:10.1136/bmjopen-2024-092494)
Supplement: online supplemental material 2 [file bmjopen-15-6-s002.docx]

**Supplementary material 2**

**Supplementary A. Search strategy and terms**

**EMBASE**

1. Initial search

| **#** | **Searches** | **Results** |
| --- | --- | --- |
| 1 | exp Salmonella/ | 63900 |
| 2 | salmonell*.mp. [mp=title, abstract, heading word, drug trade name, original title, device manufacturer, drug manufacturer, device trade name, keyword heading word, floating subheading word, candidate term word] | 127743 |
| 3 | 1 or 2 | 127743 |
| 4 | exp typhoid fever/ | 18774 |
| 5 | typhoid fever.mp. [mp=title, abstract, heading word, drug trade name, original title, device manufacturer, drug manufacturer, device trade name, keyword heading word, floating subheading word, candidate term word] | 19858 |
| 6 | 4 or 5 | 19858 |
| 7 | 3 or 6 | 138816 |
| 8 | exp "length of stay"/ | 224675 |
| 9 | (length or stay or LOS).mp. [mp=title, abstract, heading word, drug trade name, original title, device manufacturer, drug manufacturer, device trade name, keyword heading word, floating subheading word, candidate term word] | 1238504 |
| 10 | 8 or 9 | 1238504 |
| 11 | exp hospitalization/ | 447337 |
| 12 | (hospitali?ation or hospitali?ed).mp. [mp=title, abstract, heading word, drug trade name, original title, device manufacturer, drug manufacturer, device trade name, keyword heading word, floating subheading word, candidate term word] | 686777 |
| 13 | 11 or 12 | 686777 |
| 14 | exp "cost"/ | 377137 |
| 15 | (cost or costs).mp. [mp=title, abstract, heading word, drug trade name, original title, device manufacturer, drug manufacturer, device trade name, keyword heading word, floating subheading word, candidate term word] | 1111616 |
| 16 | 14 or 15 | 1125671 |
| 17 | 10 or 13 or 16 | 2733045 |
| 18 | exp drug resistance/ | 353336 |
| 19 | resistan*.mp. [mp=title, abstract, heading word, drug trade name, original title, device manufacturer, drug manufacturer, device trade name, keyword heading word, floating subheading word, candidate term word] | 1732277 |
| 20 | 18 or 19 | 1732277 |
| 21 | exp antiinfective agent/ | 4161512 |
| 22 | (antibiotic drug or antimicrobial drug or fluoroquinolone or carbapenem or ciprofloxacin or ampicillin or co?trimoxazole or tetracycline or cephalosporin or trimethoprim or chloramphenicol or azithromycin or aminoglycoside or piperacillin or tazobactam or erythromycin or doxycycline or furazolidone or metronidazole or vancomycin or fidaxomicin).mp. [mp=title, abstract, heading word, drug trade name, original title, device manufacturer, drug manufacturer, device trade name, keyword heading word, floating subheading word, candidate term word] | 641074 |
| 23 | 21 or 22 | 4189768 |
| 24 | 7 and 17 | 6993 |
| 25 | 20 and 24 | 1685 |
| 26 | 23 and 24 | 2535 |
| 27 | 20 and 23 and 24 | 1257 |
| 28 | exp animals/ or exp invertebrate/ or animal experiment/ or animal model/ or animal tissue/ or animal cell/ | 30607956 |
| 29 | exp human/ or exp "human tissue, cells or cell components"/ | 24486569 |
| 30 | 28 and 29 | 24398593 |
| 31 | 28 not 30 | 6209363 |
| 32 | 24 not 31 | 6132 |
| 33 | limit 32 to dc=20050101-20211217 | 4438 |
| 34 | 25 not 31 | 1488 |
| 35 | limit 34 to dc=20050101-20211217 | 1135 |

1. Search updates

| **#** | **Searches** | **Results** |
| --- | --- | --- |
| 1 | exp Salmonella/ | 74890 |
| 2 | salmonell*.mp. | 140413 |
| 3 | 1 or 2 | 140413 |
| 4 | exp typhoid fever/ | 20146 |
| 5 | typhoid fever.mp. | 21338 |
| 6 | 4 or 5 | 21338 |
| 7 | 3 or 6 | 152233 |
| 8 | exp "length of stay"/ | 290462 |
| 9 | (length or stay or LOS).mp. | 1455967 |
| 10 | 8 or 9 | 1455967 |
| 11 | exp hospitalization/ | 579620 |
| 12 | (hospitali?ation or hospitali?ed).mp. | 868544 |
| 13 | 11 or 12 | 868544 |
| 14 | exp "cost"/ | 424052 |
| 15 | (cost or costs).mp. | 1308317 |
| 16 | 14 or 15 | 1322863 |
| 17 | 10 or 13 or 16 | 3252662 |
| 18 | exp drug resistance/ | 407036 |
| 19 | resistan*.mp. | 2016720 |
| 20 | 18 or 19 | 2016720 |
| 21 | exp antiinfective agent/ | 5113394 |
| 22 | (antibiotic drug or antimicrobial drug or fluoroquinolone or carbapenem or ciprofloxacin or ampicillin or co?trimoxazole or tetracycline or cephalosporin or trimethoprim or chloramphenicol or azithromycin or aminoglycoside or piperacillin or tazobactam or erythromycin or doxycycline or furazolidone or metronidazole or vancomycin or fidaxomicin).mp. | 735271 |
| 23 | 21 or 22 | 5144171 |
| 24 | 7 and 17 | 8232 |
| 25 | 20 and 24 | 2114 |
| 26 | 23 and 24 | 3351 |
| 27 | 20 and 23 and 24 | 1603 |
| 28 | exp animals/ or exp invertebrate/ or animal experiment/ or animal model/ or animal tissue/ or animal cell/ | 34592509 |
| 29 | exp human/ or exp "human tissue, cells or cell components"/ | 28042510 |
| 30 | 28 and 29 | 27945650 |
| 31 | 28 not 30 | 6646859 |
| 32 | 24 not 31 | 7211 |
| 33 | limit 32 to dc=20050101-20211217 | 4410 |
| 34 | 25 not 31 | 1857 |
| 35 | limit 34 to dc=20211215-20240515 | 374 |

**MEDLINE**

1. Initial search

| **#** | **Searches** | **Results** |
| --- | --- | --- |
| 1 | exp Salmonella/ | 67144 |
| 2 | salmonell*.mp. [mp=title, abstract, original title, name of substance word, subject heading word, floating sub-heading word, keyword heading word, organism supplementary concept word, protocol supplementary concept word, rare disease supplementary concept word, unique identifier, synonyms] | 98329 |
| 3 | 1 or 2 | 98329 |
| 4 | exp typhoid fever/ | 11351 |
| 5 | typhoid fever.mp. [mp=title, abstract, original title, name of substance word, subject heading word, floating sub-heading word, keyword heading word, organism supplementary concept word, protocol supplementary concept word, rare disease supplementary concept word, unique identifier, synonyms] | 13374 |
| 6 | 4 or 5 | 13374 |
| 7 | 3 or 6 | 106591 |
| 8 | exp "length of stay"/ | 97372 |
| 9 | (length or stay or LOS).mp. [mp=title, abstract, original title, name of substance word, subject heading word, floating sub-heading word, keyword heading word, organism supplementary concept word, protocol supplementary concept word, rare disease supplementary concept word, unique identifier, synonyms] | 910890 |
| 10 | 8 or 9 | 910890 |
| 11 | exp hospitalization/ | 271126 |
| 12 | (hospitali?ation or hospitali?ed).mp. [mp=title, abstract, original title, name of substance word, subject heading word, floating sub-heading word, keyword heading word, organism supplementary concept word, protocol supplementary concept word, rare disease supplementary concept word, unique identifier, synonyms] | 334914 |
| 13 | 11 or 12 | 459952 |
| 14 | exp "cost"/ | 252633 |
| 15 | (cost or costs).mp. [mp=title, abstract, original title, name of substance word, subject heading word, floating sub-heading word, keyword heading word, organism supplementary concept word, protocol supplementary concept word, rare disease supplementary concept word, unique identifier, synonyms] | 722337 |
| 16 | 14 or 15 | 734607 |
| 17 | 10 or 13 or 16 | 1892772 |
| 18 | exp drug resistance/ | 356150 |
| 19 | resistan*.mp. [mp=title, abstract, original title, name of substance word, subject heading word, floating sub-heading word, keyword heading word, organism supplementary concept word, protocol supplementary concept word, rare disease supplementary concept word, unique identifier, synonyms] | 1231174 |
| 20 | 18 or 19 | 1231174 |
| 21 | exp Anti-Bacterial Agents/ | 771169 |
| 22 | (antibiotic drug or antimicrobial drug or fluoroquinolone or carbapenem or ciprofloxacin or ampicillin or co?trimoxazole or tetracycline or cephalosporin or trimethoprim or chloramphenicol or azithromycin or aminoglycoside or piperacillin or tazobactam or erythromycin or doxycycline or furazolidone or metronidazole or vancomycin or fidaxomicin).mp. [mp=title, abstract, original title, name of substance word, subject heading word, floating sub-heading word, keyword heading word, organism supplementary concept word, protocol supplementary concept word, rare disease supplementary concept word, unique identifier, synonyms] | 264162 |
| 23 | 21 or 22 | 847941 |
| 24 | 7 and 17 | 4441 |
| 25 | 20 and 24 | 977 |
| 26 | 23 and 24 | 909 |
| 27 | 20 and 23 and 24 | 600 |
| 28 | exp animals/ not humans.sh. | 4932653 |
| 29 | 27 not 28 | 536 |
| 30 | (2005* or 2006* or 2007* or 2008* or 2009* or 201* or 2020* or 20210* or 202110* or 202111* or 2021120* or "20211210" or "20211211" or "20211212" or "20211213" or "20211214" or "20211215" or "20211216" or "20211217").dt,ez,ed. | 18420885 |
| 31 | 29 and 30 | 362 |

1. Search updates

| **#** | **Searches** | **Results** |
| --- | --- | --- |
| 1 | exp Salmonella/ | 70144 |
| 2 | salmonell*.mp. | 105479 |
| 3 | 1 or 2 | 105479 |
| 4 | exp typhoid fever/ | 11762 |
| 5 | typhoid fever.mp. | 14269 |
| 6 | 4 or 5 | 14269 |
| 7 | 3 or 6 | 114245 |
| 8 | exp "length of stay"/ | 104709 |
| 9 | (length or stay or LOS).mp. | 1042547 |
| 10 | 8 or 9 | 1042547 |
| 11 | exp hospitalization/ | 301351 |
| 12 | (hospitali?ation or hospitali?ed).mp. | 405068 |
| 13 | 11 or 12 | 541137 |
| 14 | exp "cost"/ | 270579 |
| 15 | (cost or costs).mp. | 860354 |
| 16 | 14 or 15 | 875816 |
| 17 | 10 or 13 or 16 | 2215640 |
| 18 | exp drug resistance/ | 383612 |
| 19 | resistan*.mp. | 1428024 |
| 20 | 18 or 19 | 1428024 |
| 21 | exp Anti-Bacterial Agents/ | 837948 |
| 22 | (antibiotic drug or antimicrobial drug or fluoroquinolone or carbapenem or ciprofloxacin or ampicillin or co?trimoxazole or tetracycline or cephalosporin or trimethoprim or chloramphenicol or azithromycin or aminoglycoside or piperacillin or tazobactam or erythromycin or doxycycline or furazolidone or metronidazole or vancomycin or fidaxomicin).mp. | 293540 |
| 23 | 21 or 22 | 927070 |
| 24 | 7 and 17 | 4952 |
| 25 | 20 and 24 | 1137 |
| 26 | 23 and 24 | 1046 |
| 27 | 20 and 23 and 24 | 697 |
| 28 | exp animals/ not humans.sh. | 5223076 |
| 29 | 27 not 28 | 620 |
| 34 | ("20211215" or "20211216" or "20211217" or "20211218" or "20211219" or 2021122* or 2021123* or 2022* or 2023* or 202401* or 202402* or 202403* or 202404* or 2024050* or "20250510" or "20240511" or "20240512" or "20250513" or "20240514" or "20240515").dt,ez,ed. | 4249365 |
| 35 | 29 and 34 | 91 |

**SCOPUS**

- Initial search & Search updates

(salmonell* OR “typhoid fever”) AND (“length of stay” OR length or stay or LOS OR hospitali?ation OR hospitali?ed OR cost*) AND (resistan*)

**Hinari (research for life)**

- Initial search & Search updates

((Abstract:(salmonell* OR “typhoid fever”)) AND ((Abstract:("length of stay")) OR (Abstract:(los)) OR (Abstract:(length)) OR (Abstract:(stay)) OR (Abstract:(hospitali*ation)) OR (Abstract:(hospitali*ed)) OR (Abstract:(cost))) AND (Abstract:(resistan*)).

**LILACS**

- Initial search & Search updates

((SALMONELLA) OR (TYPHOID)) AND ((LENGTH) OR (STAY) OR (HOSPITALIZATION) OR (HOSPITALIZED) OR (HOSPITALISED) OR (HOSPITALISATION) OR (COST)) AND ((RESISTANT) OR (RESISTANCE) OR (‘DRUG RESISTANT’))

**Supplementary B**. PICO (patient/population, intervention, comparison and outcomes) framework and inclusion/exclusion criteria

- Exclude studies where data were collected prior to 2005
- No exclusion criteria based on language
- This study will focus on hospital costs, but some community cost estimates may also be gathered
- Minimum 30 patients per pathogen and study

| **PICO definitions** | **Inclusion** | **Exclusion** |
| --- | --- | --- |
| Population | Humans infected with antimicrobial-resistant and/or susceptible bacteria: typhoidal *Salmonella* (*Salmonella enterica* Typhi and Paratyphi) and non-typhoidal *Salmonella*. | Animals |
| Intervention | Not applicable | Not applicable |
| Comparison | Length of stay estimates were pooled for susceptible and drug-resistant infections from the different studies, even if individual studies only focused on susceptible or drug-resistant infections. |  |
| Outcome | Cost per case; Length of hospital stay, (may additionally include indicators of community cost where available, e.g. length of illness) |  |
| Study design | Observational studies (cohort, case-control, cross-sectional, and case series), randomised controlled trials (RCTs), modelling studies, and economic evaluations (including cost-of-illness studies) | Editorials, reviews, commentaries, conference abstracts, case reports |

**Supplementary C.** JBI’s critical appraisal tools

| Research design | Questions |
| --- | --- |
| Case series study | - Were there clear criteria for inclusion in the case series? - Was the condition measured in a standard, reliable way for all participants included in the case series? - Were valid methods used for identification of the condition for all participants included in the case series? - Did the case series have consecutive inclusion of participants? - Did the case series have complete inclusion of participants? - Was there clear reporting of the demographics of the participants in the study? - Was there clear reporting of clinical information of the participants? - Were the outcomes or follow up results of cases clearly reported? - Was there clear reporting of the presenting site(s)/clinic(s) demographic information? - Was statistical analysis appropriate? |
| Cohort study | - Were the two groups similar and recruited from the same population? - Were the exposures measured similarly to assign people to both exposed and unexposed groups? - Was the exposure measured in a valid and reliable way? - Were confounding factors identified? - Were strategies to deal with confounding factors stated? - Were the groups/participants free of the outcome at the start of the study (or at the moment of exposure)? - Were the outcomes measured in a valid and reliable way? - Was the follow up time reported and sufficient to be long enough for outcomes to occur? - Was follow up complete, and if not, were the reasons to loss to follow up described and explored? - Were strategies to address incomplete follow up utilized? - Was appropriate statistical analysis used? |
| Prevalence study | - Was the sample frame appropriate to address the target population? - Were study participants sampled in an appropriate way? - Was the sample size adequate? - Were the study subjects and the setting described in detail? - Was the data analysis conducted with sufficient coverage of the identified sample? - Were valid methods used for the identification of the condition? - Was the condition measured in a standard, reliable way for all participants? - Was there appropriate statistical analysis? - Was the response rate adequate, and if not, was the low response rate managed appropriately? |
| Cost of illness study**†** | - Is the scope of the economic burden well defined with a clear indication of the study perspective? - Are the disease and target population clearly described? - Are all cost categories and components clearly defined and described? - Are all important and relevant cost components comprehensively included based on the study perspective? - Are the data sources used to estimate disease burden and costs clearly described? - Are costs measured accurately? - Are costs valued credibly? - Are costs adjusted for differential timing? - Was appropriate statistical analysis used? - Were sensitivity analyses conducted to investigate uncertainty in cost estimates? - Are generalizability of results adequately discussed? |
| Randomized Controlled Trials (RCTs) | - Was true randomization used for assignment of participants to treatment groups? - Was allocation to treatment groups concealed? - Were treatment groups similar at the baseline? - Were participants blind to treatment assignment? - Were those delivering the treatment blind to treatment assignment? - Were treatment groups treated identically other than the intervention of interest? - Were outcome assessors blind to treatment assignment? - Were outcomes measured in the same way for treatment groups? - Were outcomes measured in a reliable way - Was follow up complete and if not, were differences between groups in terms of their follow up adequately described and analysed? - Were participants analysed in the groups to which they were randomized? - Was appropriate statistical analysis used? - Was the trial design appropriate and any deviations from the standard RCT design (individual randomization, parallel groups) accounted for in the conduct and analysis of the trial? |

**†** The JBI critical appraisal tool does not include tools for assessing cost-of-illness studies. Therefore, an adapted version is used, incorporating the JBI tool designed for economic evaluations.

The checklist comprises questions with “yes,” “no,” “unclear,” or “not applicable” responses. We calculated quality scores between 0 and 1 by dividing the number of “yes” responses by the total number of “yes,” “no,” and “unclear” responses, while “Not applicable” responses were excluded. This approach to quality scoring is consistent with the method employed in other studies, where the score is derived as the proportion of applicable checklist criteria met [(1)](https://sciwheel.com/work/citation?ids=5173387&pre=&suf=&sa=0).

**Supplementary D.** Methods for Conversion and Meta-Analysis

For monetary costs, we adopted the previous code developed [(2,3)](https://sciwheel.com/work/citation?ids=15742678,16119103&pre=&pre=&suf=&suf=&sa=0,0) to convert the costs in the included studies to 2019 United States dollars (USD) to make the costs for different years and countries comparable. For monetary cost data originally reported in local currencies, we first inflated the local currency amount to 2019 using the World Bank Group GDP deflators for that economy [(4)](https://sciwheel.com/work/citation?ids=15714088&pre=&suf=&sa=0). This helped account for inflation in the local economy. We then converted the inflation-adjusted 2019 local currency amount to 2019 US dollars (USD) using the relevant 2019 exchange rates from the World Bank [(5)](https://sciwheel.com/work/citation?ids=15714442&pre=&suf=&sa=0). This two-step process allowed us to infer what the monetary costs would be in 2019 USD. For costs already reported in USD, we first had to convert them back to the local currency amounts from the year of the study using exchange rates of the respective year [(5)](https://sciwheel.com/work/citation?ids=15714442&pre=&suf=&sa=0). This provided the costs in local currency without adjusting for inflation over the years. We could then follow the same two-step approach described above to inflate the local currency amount to 2019 levels using the GDP deflators and finally convert to 2019 USD using the 2019 exchange rates [(6)](https://sciwheel.com/work/citation?ids=9009534&pre=&suf=&sa=0).

We conducted conversions and data synthesis using R software (version 4.2.3) and the *metamean* function [(7)](https://sciwheel.com/work/citation?ids=3287388&pre=&suf=&sa=0). For all extracted data, including the number of days and costs, we converted any estimations in other measures (i.e. median) and uncertainty/range values (i.e. standard deviation, range, interquartile range) into means and 95% confidence intervals to make results comparable across studies. We performed the meta-analyses using a random effects model at the quantile level of GDP per capita [(8)](https://sciwheel.com/work/citation?ids=17388924&pre=&suf=&sa=0), including only subcategories with more than three estimates from different study points.

**Supplementary E.** Duration of fever and length of hospital stay for patients with susceptible and antibiotic-resistant *Salmonella* infections

| Question | Pathogen | Classification | Country | Quantile level of GDP per capita | Sample size | Value (95% CI)†§ | Reference |
| --- | --- | --- | --- | --- | --- | --- | --- |
| What is the length of a symptomatic infection with a drug-susceptible, mixed or unknown resistance pathogen?  (fever clearance time) | **S. Typhi and/or S. Paratyphi** | **Pooled** |  | **1** |  | **11.7 (3.3-20)** |  |
|  | S. Typhi | Nalidixic acid sus. | India | 1 | 4 | 4.8 (0-9.8) | Bandyopadhyay et al. |
|  | S. Typhi | Drug-sensitive | Pakistan | 1 | 13 | 26.6 (12.4-40.8) | Herekar et al. |
|  | S. Typhi | Drug-sensitive | Pakistan | 1 | 31 | 16 (8.4-23.6) | Herekar et al. |
|  | S. Typhi and S. Paratyphi | Not reported | India | 1 | 30 | 6.4 (5-7.8) | Dahiya et al. |
|  | S. Typhi and S. Paratyphi | Nalidixic acid sus. | Australia | 4 | 57 | 3.3 | Hume et al. |
|  | S. Typhi | Mixed | Australia | 4 | 59 | 4.7 | Hume et al. |
|  | S. Paratyphi | Mixed | Australia | 4 | 45 | 3.1 | Hume et al. |
|  | S. non-Typhi | Mixed | Taiwan | 4 | 67 | 4.7 (4-5.4) | Lee et al. |
| What is the length of a symptomatic infection with a drug resistance pathogen?  (fever clearance time) | **S. Typhi and/or S. Paratyphi** | **Pooled** |  | **1** |  | **13.7 (8.1-19.2)** |  |
|  | S. Typhi | MDR | India | 1 | 8 | 8.7 (4.5-13) | Bandyopadhyay et al. |
|  | S. Typhi | MDR | Pakistan | 1 | 77 | 24.6 (16.3-32.9) | Herekar et al. |
|  | S. Typhi | MDR | Pakistan | 1 | 629 | 10 (7.4-12.7) | Herekar et al. |
|  | S. Typhi | XDR | Pakistan | 1 | 86 | 18.8 (14.9-22.6) | Herekar et al. |
|  | S. Typhi | XDR | Pakistan | 1 | 679 | 9.5 (7.7-11.3) | Herekar et al. |
|  | S. Typhi | MDR | Turkey | 3 | 137 | 3.2 (3-3.4) | Aypak et al. |
|  | S. Typhi | MDR | Turkey | 3 | 15 | 4.6 (3.7-5.5) | Aypak et al. |
|  | S. Typhi and S. Paratyphi | Nalidixic acid res. | Australia | 4 | 39 | 5.6 | Hume et al. |
| What is the length of hospitalisation with a drug-susceptible, mixed or unknown resistance pathogen? | **S. Typhi and/or S. Paratyphi** | **Pooled** |  | **1** |  | **6.4 (4.9-7.8)** |  |
|  | S. Typhi | Drug-sensitive | Pakistan | 1 | 13 | 4 (3.3-4.7) | Herekar et al. |
|  | S. Typhi | Drug-sensitive | Pakistan | 1 | 31 | 5 (4.2-5.7) | Herekar et al. |
|  | S. Typhi and S. Paratyphi | Mixed | Bangladesh | 1 | 1295 | 6.7 (6.5-6.9) | Longley et al. |
|  | S. Typhi | Mixed | India | 1 | 303.36 | 6.5 | Ganesh et al. |
|  | S. Typhi and S. Paratyphi | Mixed | India | 1 | 156 | 6 (5.8-6.2) | Sharma et al. |
|  | S. Typhi and S. Paratyphi | Mixed | Nepal | 1 | 455 | 6 (5.7-6.3) | Longley et al. |
|  | S. Typhi and S. Paratyphi | Mixed | Pakistan | 1 | 1054 | 3.4 (3.2-3.5) | Longley et al. |
|  | S. Typhi and S. Paratyphi | Not reported | India | 1 | 146 | 9.6 (8.8-10.5) | Bandyopadhyay et al. |
|  | S. Typhi and S. Paratyphi | Not reported | India | 1 | 30 | 10 (7.1-12.9) | Dahiya et al. |
|  | S. Typhi and S. Paratyphi | Not reported | India | 1 | 63 | 7.4 | Nagaraj et al. |
|  | S. Typhi and S. Paratyphi | Not reported | India | 1 | 63 | 9.4 | Nagaraj et al. |
|  | S. Typhi | Non-XDR | Pakistan | 1 | 505 | 7.9 (7.1-8.7) | Fatima et al. |
|  | S. Typhi and S. Paratyphi | Nalidixic acid sus. | Australia | 4 | 57 | 5.7 | Hume et al. |
|  | S. Typhi | Mixed | Australia | 4 | 59 | 6.7 | Hume et al. |
|  | S. Paratyphi | Mixed | Australia | 4 | 45 | 5.8 | Hume et al. |
|  | S. Typhi and S. Paratyphi | Mixed | New Zealand | 4 | 162 | 6 (5.5-6.5) | Lane et al. |
|  | S. non-Typhi | Mixed | Vietnam | 2 | 450 | 5 (4.7-5.3) | Duong et al. |
|  | S. non-Typhi | Nalidixic acid sus. | China | 3 | 163 | 4.7 | Broughton et al. |
|  | S. non-Typhi | Mixed | China | 3 | 171 | 6.1 (5.4-6.7) | Liang et al. |
|  | **S. non-Typhi** | **Pooled** |  | **4** |  | **6.7 (5.6-7.8)** |  |
|  | S. non-Typhi | Pansusceptible | USA | 4 | 48 | 4.0 | Mukherjee et al. |
|  | S. non-Typhi | Pansusceptible | USA | 4 | 301 | 5.2 (4.2-6.2) | Solghan et al. |
|  | S. non-Typhi | Ampicillin sus. | USA | 4 | 49 | 4.0 | Mukherjee et al. |
|  | S. non-Typhi | Tetracycline sus. | USA | 4 | 52 | 4.2 | Mukherjee et al. |
|  | S. non-Typhi | Mixed | Italy | 4 | 252 | 5.7 (5.1-6.3) | Pagani et al. |
|  | S. non-Typhi | Mixed | Taiwan | 4 | 118 | 6.8 (6.2-7.4) | Huang et al. |
|  | S. non-Typhi | Mixed | Taiwan | 4 | 179 | 8.6 (7.8-9.4) | Huang et al. |
|  | S. non-Typhi | Not reported | Spain | 4 | 21660 | 7.1 (7-7.2) | Garrido-Estepa et al. |
| What is the length of hospitalisation with a drug resistance pathogen? | **S. Typhi** | **Pooled** |  | **1** |  | **8.4 (5.1-11.7)** |  |
|  | S. Typhi | MDR | Pakistan | 1 | 77 | 5.4 (4.8-6) | Herekar et al. |
|  | S. Typhi | MDR | Pakistan | 1 | 629 | 4.1 (3.9-4.3) | Herekar et al. |
|  | S. Typhi | XDR | Pakistan | 1 | 947 | 12.3 (11.2-13.4) | Fatima et al. |
|  | S. Typhi | XDR | Pakistan | 1 | 101 | 6 (5.1-6.9) | Shahid et al. |
|  | S. Typhi | XDR | Pakistan | 1 | 86 | 8.4 (7.6-9.2) | Herekar et al. |
|  | S. Typhi | XDR | Pakistan | 1 | 679 | 6.1 (5.9-6.3) | Herekar et al. |
|  | S. Typhi | XDR | Pakistan | 1 | 77 | 16.6 (15.7-17.4) | Khan et al. |
|  | S. Typhi | Amikacin res. | Turkey | 3 | 45 | 2.2 (1.9-2.5) | Karakecili et al. |
|  | S. Typhi | MDR | Turkey | 3 | 137 | 3.7 (3.4-4) | Aypak et al. |
|  | S. Typhi | MDR | Turkey | 3 | 15 | 5.6 (4.5-6.7) | Aypak et al. |
|  | S. Typhi and S. Paratyphi | Nalidixic acid res. | Australia | 4 | 39 | 7.9 | Hume et al. |
|  | S. non-Typhi | Nalidixic acid res. | China | 3 | 162 | 8.5 | Broughton et al. |
|  | S. non-Typhi | MDR | China | 3 | 49 | 8.9 (6.6-11.3) | Liang et al. |
|  | S. non-Typhi | Ampicillin res. | USA | 4 | 10 | 6.2 | Mukherjee et al. |
|  | S. non-Typhi | res. | USA | 4 | 39 | 5.9 | Mukherjee et al. |
|  | S. non-Typhi | Tetracycline res. | USA | 4 | 7 | 6.0 | Mukherjee et al. |
|  | S. non-Typhi | MDR | USA | 4 | 79 | 5.7 (4.6-6.8) | Solghan et al. |

† Mean values (how other forms of values were transformed to mean and confidence intervals; <https://rdrr.io/cran/meta/man/metamean.html>)

§ Length of a symptomatic infection and hospitalisation are in days

Monetary Values are in 2019 USD

[**Bibliography**](https://sciwheel.com/work/bibliography)

[1. Naylor NR, Atun R, Zhu N, Kulasabanathan K, Silva S, Chatterjee A, et al. Estimating the burden of antimicrobial resistance: a systematic literature review. Antimicrob Resist Infect Control. 2018 Apr 25;7:58.](https://sciwheel.com/work/bibliography/5173387)

[2. Naylor NR. The Antimicrobial Resistance Unit Cost Repository (AMR-UCR) [Internet]. GitHub. [cited 2023 Sep 1]. Available from: https://github.com/NikkiR08/AMR-UCR](https://sciwheel.com/work/bibliography/15742678)

[3. Naylor NR, Hasso-Agopsowicz M, Kim C, Ma Y, Frost I, Abbas K, et al. The global economic burden of antibiotic resistant infections and the potential impact of bacterial vaccines: A modelling study. 2023;](https://sciwheel.com/work/bibliography/16119103)

[4. World Bank Group. GDP deflator (base year varies by country) [Internet]. [cited 2023 Nov 29]. Available from: https://data.worldbank.org/indicator/NY.GDP.DEFL.ZS](https://sciwheel.com/work/bibliography/15714088)

[5. World Bank Group. Official exchange rate (LCU per US$, period average) [Internet]. [cited 2023 Nov 29]. Available from: https://data.worldbank.org/indicator/PA.NUS.FCRF](https://sciwheel.com/work/bibliography/15714442)

[6. Turner HC, Lauer JA, Tran BX, Teerawattananon Y, Jit M. Adjusting for inflation and currency changes within health economic studies. Value Health. 2019;22(9):1026–32.](https://sciwheel.com/work/bibliography/9009534)

[7. Schwarzer G, Carpenter JR, Rücker G. Meta-Analysis with R. Cham: Springer International Publishing; 2015.](https://sciwheel.com/work/bibliography/3287388)

[8. World Bank Group. GDP per capita (current US$) [Internet]. [cited 2025 Jan 11]. Available from: https://data.worldbank.org/indicator/NY.GDP.PCAP.CD](https://sciwheel.com/work/bibliography/17388924)
