# Supplementary material for: Length of hospital stay and associated treatment costs for patients with susceptible and antibiotic-resistant Salmonella infections: a systematic review and meta-analysis
Source: BMJ Open. 2025 Jun 23;15(6):e092494. doi: 10.1136/bmjopen-2024-092494 (PMC12186044; doi:10.1136/bmjopen-2024-092494)
Supplement: Supplementary file 3 [file bmjopen-2024-092494.reviewer_comments.pdf]

PEER REVIEW HISTORY

BMJ Open publishes all reviews undertaken for accepted manuscripts. Reviewers are asked to complete a checklist review form and are provided with free text boxes to elaborate on their assessment. These free text comments are reproduced below.

ARTICLE DETAILS

Title (Provisional)

Length of hospital stay and associated treatment costs for patients with susceptible and antibiotic-resistant Salmonella infections: a systematic review and meta-analysis

Authors

Kim, Chaelin; Frost, Isabel; Naylor, Nichola R; Au, Heidi; Kim, Yeonhee; Lee, Yubin; Bzymek, Anna; Majgier, Kamila; Moldoveanu, Ana Laura; Salman, Omar Mukhtar; Simiyu, Shillah; Youssef, Dina Mohamed; Hasso-Agopsowicz, Mateusz; Abbas, Kaja

---

VERSION 1 - REVIEW

**Reviewer**	**1**
**Name**	**Moinuddin, Mohammed**
**Affiliation**	**University of Central Lancashire, School of Medicine and Dentistry**
**Date**	**07-Feb-2025**
**COI**	**None**

Statistical reviewer’s comment

Results from a systematic review and meta-analysis to estimate the length of hospital stay and associated treatment costs for patients with susceptible or antibiotic-resistant Salmonella Typhi, Paratyphi, and non-typhoidal Salmonella infections. An interesting and valuable piece of research however the methodology and statistical analysis plan required further clarification and justification. My observations are below:

Comment 1 – The authors said that the PRISMA guidelines to conduct the systematic review. It is to be noted that PRISMA is the guideline to report the systematic review not to conduct it. To conduct a systematic review the appropriate guideline is the Cochrane Handbook of Systematic Review (Chandler et al, 2019).

Comment 2 – The authors said that the search was conducted in two stages for different dates. I wonder what the reason for that was and most importantly was the same search strategy applied in both stages.

Comment 3 – The authors used the DistillerSR which is a Machine Learning based auto screening tool. However, there is no description of how the training sets were prepared and what relevance thresholds were applied in screening the articles. Also, care must be taken to interpret the results as the sensitivity of DistillerSR is not very high. A description of the full implementation of the tool is required. Also, provide the reference.

Comment 4 – A random effect meta-analysis was applied to combine the results. I wonder whether the choice was made by any statistical consideration for example checking the heterogeneity.

Comment 5 – The meta-analyses were presented by wealth quintile (forest plot). A summary of the methods for constructing the wealth quintile is required especially what components were included in the wealth index. Moreover, the Principal component analysis based wealth quintile is the comparative measure so it is not possible to compare the quintile bases by country. How do the authors account for this issue in interpreting the results?

Reference

Chandler, J., Cumpston, M., Li, T., Page, M. J., & Welch, V. J. H. W. (2019). Cochrane handbook for systematic reviews of interventions. Hoboken: Wiley.

Reviewer 2

Name Pires, Sara

Affiliation National Food Institute, Technical University of Denmark , Division of Diet, Toxicology and Disease Prevention

Date 19-Feb-2025

COI None

The manuscript describes an interesting and well-designed study that addresses an important knowledge gap. It is well-structured and written, and generates results that will be useful to understand the health and economic burden of resistant Salmonella infections. The study's results will be important to quantify the burden of AMR Salmonella, which is crucial to provide evidence for the urgent prevention of AMR. The introduction and methods sections are clear and comprehensive (while succinct).

My main comments are on the description of the results:

- While the description of the results is thorough, I feel that the results of meta-analysis should be better described. The text provides a good description of the individual studies' result (i.e. extracted data), but it would be interesting for the reader to understand what the pooled analysis of all these estimates shows first. This goes for length of hospital stays, duration of fever, and costs. The supplements show the pooled results in bold, which is excellent, but it would be good to have a more structured presentation in the text

- Aligned with this, there seems to be no reference to figures 2 and 3 in the text. Those figures show the results of the random effects model, which are interesting for the reader

- For a better flow, the authors could consider presenting the results of costs last (after the health parameters)

- For the costs' estimates, I think it would also be interesting to see the mean costs estimated using all extracted data, and then the description of the individual studies.

- The discussion reads and all points raised are important. I feel the authors could expand on how the estimates they generated will be important to contribute to the disease and economic burden of AMR Salmonella, and could pave the way to do the same (or call for more research) for other pathogens. For example, disease models used for DALY calculations will benefit from these estimates.

Minor comments:

- Under "what this study adds", it is unclear why the authors focus on typhind endemic data gaps only (second highlight)

- Under "how this stud might affect research, practice and policy", I am unsure if the findings highlight the economic burden (…), or if they provide evidence to estimate the economic burden – which has many more layers and contributors.

- Lines 101-104: this sentence was difficult to read. Suggest rephrasing

- Line 146: I suggest adding that you extracted data on the type of antimicrobial drug used

---

VERSION 1 - AUTHOR RESPONSE

Reviewer #1

Dr. Mohammed Moinuddin, University of Central Lancashire
Comments to the Author:
Statistical reviewer's comment
Results from a systematic review and meta-analysis to estimate the length of hospital stay and associated treatment costs for patients with susceptible or antibiotic-resistant Salmonella Typhi, Paratyphi, and non-typhoidal Salmonella infections. An interesting and valuable piece of research however the methodology and statistical analysis plan required further clarification and justification. My observations are below:

Comment 1 – The authors said that the PRISMA guidelines to conduct the systematic review. It is to be noted that PRISMA is the guideline to report the systematic review not to conduct it. To conduct a systematic review the appropriate guideline is the Cochrane Handbook of Systematic Review (Chandler et al, 2019).

- Thank you for highlighting this point. We agree that PRISMA serves as a guideline for reporting systematic reviews and meta-analyses rather than their conduct. Accordingly, we have revised the sentence at the start of the 'Search strategy' section to reflect this distinction. The revised sentence now reads:
    - "We adhered to the PRISMA (Preferred Reporting Items for Systematic Reviews and Meta-Analyses) guidelines for reporting our systematic review and meta-analysis (see **supplementary 1**)."

Comment 2 – The authors said that the search was conducted in two stages for different dates. I wonder what the reason for that was and most importantly was the same search strategy applied in both stages.

- Thank you for this important query. The search was conducted in two stages for practical reasons related to the project timeline. The first search (January 2005 - December 2021) was initially performed to feed into the economic burden analysis component of a WHO-led value attribution framework for vaccines against antimicrobial resistance, which had specific project deadlines. Subsequently, we updated the search (December 2021 - May 2024) to ensure the manuscript contained the most current evidence before journal submission. We confirm that identical search strategies were applied across both stages to maintain methodological consistency and ensure comprehensive literature coverage.
    - We conducted our search in two stages. First, we searched for articles published between 1 January 2005 and 17 December 2021, and then we updated our search to cover the period between 15 December 2021 and 15 May 2024. The same search strategy was consistently applied throughout both stages of the search to ensure continuity.

Comment 3 – The authors used the DistillerSR which is a Machine Learning based auto screening tool. However, there is no description of how the training sets were prepared and what relevance thresholds were applied in screening the articles. Also, care must be taken to interpret the results as the sensitivity of DistillerSR is not very high. A description of the full implementation of the tool is required. Also, provide the reference.

- Thank you for this comment. We would like to clarify that while DistillerSR was used as our data management platform, we did not employ its machine learning or auto-screening capabilities. As described, the team of eight reviewers conducted all article screening and data extraction manually.
    - We have updated the manuscript to clearly state that DistillerSR was used solely as a platform for manual data management and extraction: "The eight reviewers manually extracted data using standardised forms within the DistillerSR platform and used Google Sheets for subsequent data management."

Comment 4 – A random effect meta-analysis was applied to combine the results. I wonder whether the choice was made by any statistical consideration for example checking the heterogeneity.

- We employed a random-effects model specifically to account for the anticipated heterogeneity between studies. This decision was made a priori based on our understanding

of the substantial variations in healthcare systems, patient populations, clinical settings, cost calculation methodologies, and geographical contexts represented in our dataset on Salmonella and enteric infections. The random-effects approach allowed us to appropriately account for both

within-study and between-study variance

- We have added this clarification to the statistical analysis section: "A random-effects model was chosen to account for anticipated heterogeneity between studies due to differences in study designs, population characteristics, and geographical settings."

Comment 5 – The meta-analyses were presented by wealth quintile (forest plot). A summary of the methods for constructing the wealth quintile is required especially what components were included in the wealth index. Moreover, the Principal component analysis based wealth quintile is the comparative measure so it is not possible to compare the quintile bases by country. How do the authors account for this issue in interpreting the results?

- Thank you for this important methodological question. We want to clarify our methodology for constructing wealth quantiles. Rather than using a Principal Component Analysis (PCA) or composite wealth index, we categorised countries based solely on their GDP per capita from World Bank data (https://data.worldbank.org/indicator/NY.GDP.PCAP.CD). Specifically, we used 2019 GDP per capita (US$) values and created quantiles using the quantile() function in R. We acknowledge the limitation that GDP per capita as a single economic indicator does not capture all dimensions of healthcare system development. Countries within the same quantile may have different healthcare financing structures, insurance coverage, and cost calculation methodologies.
    - We have added details to the statistical analysis section: "To account for economic differences between countries, we categorised them into quartiles based on their GDP per capita using 2019 World Bank data (21). We calculated quantile cutoffs by dividing the GDP per capita values into four equal groups (quartiles), where one represented the lowest quartile and four the highest."
    - This limitation is noted in our discussion section. "Fourth, while we have pooled estimates across countries, healthcare costs can vary substantially across different healthcare systems and settings. We combined estimates to gain a broader overview of the economic burden and used random effects models along with GDP per capita quantiles to account for between-study heterogeneity and minimise differences in costs across countries. However, there remains uncertainty around applying cost estimates from one context to another."

Reference

Chandler, J., Cumpston, M., Li, T., Page, M. J., & Welch, V. J. H. W. (2019). Cochrane handbook for systematic reviews of interventions. Hoboken: Wiley.

Reviewer #2

Dr. Sara Pires, National Food Institute, Technical University of Denmark

Comments to the Author:

The manuscript describes an interesting and well-designed study that addresses an important

knowledge gap. It is well-structured and written, and generates results that will be useful to understand the health and economic burden of resistant Salmonella infections. The study's results will be important to quantify the burden of AMR Salmonella, which is crucial to provide evidence for the urgent prevention of AMR. The introduction and methods sections are clear and comprehensive (while succinct).

My main comments are on the description of the results:

- While the description of the results is thorough, I feel that the results of meta-analysis should be better described. The text provides a good description of the individual studies' result (i.e. extracted data), but it would be interesting for the reader to understand what the pooled analysis of all these estimates shows first. This goes for length of hospital stays, duration of fever, and costs. The supplements show the pooled results in bold, which is excellent, but it would be good to have a more structured presentation in the text

- Thank you for this suggestion. We have restructured our results sections to first present the pooled meta-analysis findings before discussing individual studies.

- Aligned with this, there seems to be no reference to figures 2 and 3 in the text. Those figures show the results of the random effects model, which are interesting for the reader

- We have now added references to Figures 2 and 3 in the main text.

- For a better flow, the authors could consider presenting the results of costs last (after the health parameters)

- Thank you for the suggestion. We have modified the manuscript to present the results of costs last.

- For the costs' estimates, I think it would also be interesting to see the mean costs estimated using all extracted data, and then the description of the individual studies.

- We thank the reviewer for this suggestion. We have revised the costs section to clarify: "Our meta-analysis of healthcare costs was constrained by the limited number of studies that reported costs according to resistance status. Nevertheless, the available studies consistently demonstrated higher costs related to resistant infections." We then proceed to describe the individual studies' findings.

- The discussion reads and all points raised are important. I feel the authors could expand on how the estimates they generated will be important to contribute to the disease and economic burden of AMR Salmonella, and could pave the way to do the same (or call for more research) for other pathogens. For example, disease models used for DALY calculations will benefit from these estimates.

- We thank this valuable suggestion. We have added the following sentences to the discussion section:
    - "The estimates generated in this study make a valuable contribution to understanding the overall disease and economic burden of AMR *Salmonella*. Our findings can be incorporated into disease models used for DALY calculations, allowing for more accurate assessment of the global impact of resistant infections."
    - "Robust data on the health and economic burden of resistant *Salmonella* strains

are essential for evaluating the value of these interventions. Our systematic review addresses this critical evidence gap by providing estimates that help quantify the healthcare and economic costs of AMR *Salmonella*. Furthermore, our methodological approach establishes a framework that can be applied to other high-priority pathogens where the burden of antimicrobial resistance remains poorly understood."

Minor comments:
- Under "what this study adds", it is unclear why the authors focus on typhind endemic data gaps only (second highlight)
    - We modified the sentence as follows: "A key limitation is the scarcity of data from regions with high *Salmonella* burden, particularly in sub-Saharan Africa and parts of Asia, which limits the generalisability of our findings to these high-incidence settings."
- Under "how this stud might affect research, practice and policy", I am unsure if the findings highlight the economic burden (...), or if they provide evidence to estimate the economic burden – which has many more layers and contributors.
    - As per the journal guidelines, we have removed these sections from the manuscript.
- Lines 101-104: this sentence was difficult to read. Suggest rephrasing
    - We thank the reviewer for this suggestion. We have revised the sentences as follows:
        - "Our specific study questions examine two key areas: (1) morbidity, utility, and productivity impacts (Q1-2) and (2) hospital and healthcare system costs (Q3-6)."
- Line 146: I suggest adding that you extracted data on the type of antimicrobial drug used
    - We thank the reviewer for their suggestion. However, we did not extract data on the specific type of antimicrobial drug used. Instead, we extracted and categorised data based on the antimicrobial resistance status.

VERSION 2 - REVIEW

**Reviewer**	**1**
**Name**	**Moinuddin, Mohammed**
**Affiliation**	**University of Central Lancashire, School of Medicine and Dentistry**
**Date**	**25-Apr-2025**
**COI**	

Statistical reviewer's comment

I thank the authors for taking the comments on board, clarifying and adding the explanation in the method section. The methods, especially the statistical analysis, read much clearer.

The authors verbally justified the use of the random effect model which is good but not strong scientifically. I would argue to present and interpret the random effect parameter tau-square with its significance to check if the random effect model was really necessary. Applying a random effect model extends the confidence interval and can lead to statistical insignificance of the estimated effect.

Reviewer 2

Name Pires, Sara

Affiliation National Food Institute, Technical University of Denmark , Division of Diet, Toxicology and Disease Prevention

Date 01-May-2025

COI

The authors have addressed my concerns, and the revised version of the paper is robust.

VERSION 2 - AUTHOR RESPONSE

Reviewer 1

- Statistical reviewer's comment: I thank the authors for taking the comments on board, clarifying and adding the explanation in the method section. The methods, especially the statistical analysis, read much clearer.
  - Thank you for your review and positive feedback.

- The authors verbally justified the use of the random effect model which is good but not strong scientifically. I would argue to present and interpret the random effect parameter tau-square with its significance to check if the random effect model was really necessary. Applying a random effect model extends the confidence interval and can lead to statistical insignificance of the estimated effect.
  - The significant tau-square values in our analysis represent the estimated variance of true effect sizes across studies, confirming substantial heterogeneity that supports our choice of a random effects model. This heterogeneity likely reflects genuine differences in bacterial resistance profiles, healthcare settings, and patient populations across different countries. While we acknowledge that random effects modelling produces wider confidence intervals, the random effects model is the apt model for our analysis, given the significant between-study variability observed in our data. Further, the I- squared values are high (higher than 75%) and are indicative of substantial heterogeneity.

Reviewer 2

- The authors have addressed my concerns, and the revised version of the paper is robust.
  - Thank you for your review and positive feedback.
